# Effects of combined blood flow restriction and neuromuscular electrical stimulation versus neuromuscular electrical stimulation alone on skeletal muscle hypertrophy and strength in adults: a systematic review

**DOI:** 10.3389/fphys.2026.1797485

**Published:** 2026-06-10

**Authors:** Xin Shu, Hongpeng Li, Ziyi Zhang, Xiaomei Luo, Yanli Han, Zihao Qiao, Shousheng Xu

**Affiliations:** 1 School of Sport Science, Beijing Sport University, Beijing, China; 2 Physical Education College of Shanghai University, Shanghai, China

**Keywords:** blood flow restriction, muscle strength, neuromuscular electrical stimulation, skeletal muscle hypertrophy, systematic review

## Abstract

**Objective:**

To systematically review and exploratorily analyze the effects of blood flow restriction combined with neuromuscular electrical stimulation (BFR-NMES) compared with neuromuscular electrical stimulation (NMES) alone on skeletal muscle strength and morphological adaptations in healthy adults.

**Methods:**

This systematic review was conducted in accordance with the PRISMA guidelines and registered in PROSPERO (CRD420251141357). Relevant studies published up to September 2025 were retrieved from PubMed, Web of Science, and Embase. Randomized and non-randomized controlled studies comparing BFR-NMES with NMES alone were included. Primary outcomes included muscle strength (isometric and isokinetic strength) and muscle morphology outcomes (muscle thickness, cross-sectional area, muscle mass, and thigh circumference). Risk of bias was assessed using the ROB 2.0 tool, methodological quality was evaluated using the modified Jadad scale, and evidence quality was assessed using the GRADE approach. Due to substantial clinical and methodological heterogeneity among studies, the present study primarily adopted a qualitative synthesis combined with exploratory quantitative visualization analyses.

**Results:**

Seven studies involving 124 healthy adults were included. Qualitative findings demonstrated that acute BFR-NMES interventions consistently induced greater immediate strength loss and neuromuscular fatigue, while simultaneously producing more pronounced acute muscle swelling and fluid shift responses. In contrast, long-term BFR-NMES interventions demonstrated more favorable trends in both muscle strength and muscle morphological adaptations compared with NMES alone. Exploratory forest plots further showed that acute studies generally favored NMES alone, whereas long-term studies consistently favored BFR-NMES. Collinearity analysis revealed substantial confounding between intervention duration and pressure prescription strategy: all long-term studies employed fixed-pressure protocols, whereas all acute studies adopted individualized arterial occlusion pressure (%AOP)-based strategies. No statistically significant differences were observed in the overall pooled analyses for muscle strength or rectus femoris thickness; however, substantial heterogeneity was present across studies. GRADE assessment indicated that the quality of evidence for most outcomes ranged from low to very low.

**Conclusions:**

Current evidence suggests that BFR-NMES may provide superior benefits over NMES alone in promoting acute muscle swelling and long-term muscle morphological adaptations, with potential advantages for long-term muscle strength development. However, the available evidence remains limited, and substantial confounding exists between intervention duration and pressure prescription strategies. Therefore, these findings should be interpreted cautiously. Future high-quality randomized controlled trials with larger sample sizes and independent manipulation of pressure strategies and intervention duration are warranted to clarify the true effects and optimal prescription strategies of BFR-NMES.

**Systematic review registration:**

PROSPERO, identifier: CRD420251141357.

## 1 Background

Neuromuscular electrical stimulation (NMES) is a training modality capable of inducing muscle hypertrophy and strength gains and has been widely applied in athletic training and rehabilitation. It effectively facilitates postoperative recovery, prevents disuse atrophy due to prolonged immobilization, and enhances muscle strength (Maffiuletti, 2010; Windholz et al., 2014; Leite et al., 2018; Pring et al., 2021; Başar and Alp, 2025); however, its efficacy in promoting muscular adaptations remains controversial. The underlying mechanism involves the transmission of electrical impulses to peripheral nerves, whereby greater mechanical and metabolic stress elicits involuntary muscle contractions, and the motor unit recruitment pattern differs from voluntary contractions, as NMES induces synchronous, superficial, and non-selective recruitment that bypasses the Henneman size principle, preferentially activating fast-twitch muscle fibers (Gregory and Bickel, 2005; Dasa et al., 2022; Zhou et al., 2023). Nevertheless, compared with traditional high-load resistance exercise, NMES alone is relatively limited in promoting skeletal muscle hypertrophy and strength gains, typically eliciting force levels equivalent to only 20–40% of an individual’s maximal voluntary contraction (Räuber et al., 2025).

Blood flow restriction (BFR) training creates a localized hypoxic and metabolically stressful intramuscular environment by applying external pressure to the proximal portion of the limb (Kjaer et al., 2001), representing an innovative low-load, high–metabolic stress training strategy (Macedo et al., 2025). BFR intervention alone can effectively prevent muscle atrophy during postoperative recovery or immobilization, but pronounced hypertrophic effects can only be induced when combined with active contraction training (Loenneke et al., 2012). Multiple studies have demonstrated that BFR-induced muscle hypertrophy is comparable to that achieved with 70% one-repetition maximum (1RM) load training (Suggitt et al., 2024; Hurr and Chansol, 2025). When combined with low-intensity resistance exercise (20–40% 1RM), BFR may not substantially enhance muscle strength, yet it can produce hypertrophic effects comparable to those of high-load resistance training within a short period (Ladlow et al., 2018; Lixandrão et al., 2018; Piskin et al., 2025). However, some studies have indicated that a limitation of BFR lies in its strength-training mechanism being predominantly driven by muscle hypertrophy, without inducing enhanced muscle activation or improvements in neuromuscular adaptations (Cook et al., 2013).

Based on these physiological principles, some researchers have combined BFR with NMES (BFR-NMES) as a strategy to maximize training efficiency and effectiveness. Long-term intervention studies have demonstrated that adding BFR to NMES significantly increases muscle cross-sectional area and muscle strength (Natsume et al., 2015; Gorgey et al., 2016). Studies focusing on acute effects suggest that the simultaneous application of BFR and NMES may produce synergistic effects by amplifying metabolic stress while maintaining electrically induced muscle contractions, thereby potentially eliciting superior hypertrophic and strength adaptations compared with NMES alone. Additional acute intervention studies have shown that the immediate effects of BFR-NMES manifest as muscle swelling and metabolite accumulation (Yasuda et al., 2012), whereas the long-term effects may be associated with muscle hypertrophy (Gondin et al., 2005; Natsume et al., 2015; Gorgey et al., 2016).

To date, no systematic review or meta-analysis has comprehensively and quantitatively evaluated the comparative effects of BFR-NMES versus NMES alone on muscle hypertrophy and strength in healthy adults. Therefore, the present study integrates available randomized and controlled trials in a meta-analysis to quantitatively assess the additional effects of incorporating BFR into NMES on adult muscle hypertrophy and strength outcomes. We hypothesized that, compared with NMES alone, BFR-NMES would elicit significantly greater improvements in muscle circumference and muscle strength.

## 2 Methods

This systematic review and meta-analysis was conducted in accordance with the Preferred Reporting Items for Systematic Reviews and Meta-Analyses (PRISMA) guidelines. The protocol was designed to comprehensively evaluate the comparative effects of BFR-NMES versus NMES alone on skeletal muscle hypertrophy and strength in healthy adults.

### 2.1 Literature search and screening strategy

Relevant studies were retrieved from the PubMed, Embase, and Web of Science databases, with the search period spanning from database inception to September 2025. The specific search terms included: (BFR OR Blood Flow Restriction OR Blood Flow Restriction Training OR Blood Flow Restriction Exercise) AND (EMS OR FES OR NMES OR Electric Stimulation) AND (Skeletal Muscle OR Voluntary Muscle OR Plantaris Muscle OR Soleus Muscle). Two reviewers (SX and LHP) independently screened the titles and abstracts of the retrieved studies to assess eligibility for inclusion in the meta-analysis. Any disagreements were resolved through discussion to reach consensus, with arbitration by a third reviewer (ZZY) when necessary. When information in the abstract was insufficient, the full text was reviewed for eligibility determination.

### 2.2 Protocol registration

The study protocol was registered on PROSPERO (ID: CRD420251141357).

### 2.3 Inclusion criteria

Studies were included according to the PICOS principles: (1) human trials; (2) adult participants(explicitly exclude individuals with cardiovascular diseases, neuromuscular disorders, or those taking medications that affect muscle metabolism); (3) interventions including both BFR-NMES and NMES alone; (4) pre- and post-intervention assessments of muscle strength(isometric strength, isokinetic strength); (5) pre- and post-intervention assessments of muscle hypertrophy(muscle thickness, cross-sectional area, muscle mass, thigh circumference); (6) randomized or non-randomized experimental designs; and (7) publications in English.

### 2.4 Data extraction

Two reviewers (SX and LHP) independently extracted data from all included studies. Extracted information included: (1) participant characteristics (age, sex, and BMI); (2) study characteristics (training frequency, AOP values, stimulation intensity, pressure determination methods, cuff type, and intervention duration); (3) muscle strength outcomes (isometric and isokinetic tests); and (4) muscle mass outcomes (magnetic resonance imaging or ultrasound data). When multiple time points for muscle strength or muscle mass assessments were reported, the final post-intervention measurement was used for analysis. For the purpose of this review, ‘acute interventions’ were operationally defined as protocols designed to capture the immediate physiological responses (e.g., neuromuscular fatigue, acute muscle swelling, or fluid shifts) assessed immediately post-exercise. This classification encompassed both single-session designs and multi-arm crossover trials (e.g., involving 4 or 5 sessions in total across different experimental conditions), provided that each specific intervention modality or parameter combination was administered as a single bout per condition, and the outcome assessments isolated the acute post-intervention state rather than chronic training adaptations. All data were independently recorded by both reviewers with 100% cross-checking. Any disagreements were resolved through discussion, with arbitration by a third reviewer (ZZY) when necessary. The final extracted data are presented in **Table 1**.

**Table 1:** Study characteristics.

Study	Participant groups	Demographics (age, BMI)	Number of sessions	BFR-NMES protocol	NMES protocol	Outcome measures (unit)	Jadad score
Slysz 2018 (Slysz and Burr, 2018)	10males 10females MIX(n=20)	22 ± 2y 25 ± 2kg/m^2^ 25 ± 8y 21 ± 2kg/m^2^	24	220mmHg+400μs 50-100Hz Individual Max Intensity	400μs 50-100Hz Individual Max Intensity	Maximal Isometric Strength (kg), Muscle Mass (g)	2
Li 2022 (Li et al., 2023)	20males NMES(n=10) COME(n=10)	20.8 ± 1.5y 21.3 ± 2.4kg/m^2^ 20.5 ± 1.0y 20.7 ± 3.0kg/m^2^	30	≤50cm: 200mmHg 51-55cm: 250mmHg 56-59cm: 300mmHg ≥60cm: 350mmHg+400μs 75Hz 50mA	400μs 75Hz 50mA	Maximal Isometric Strength Difference (Nm), Rectus Femoris Cross-Sectional Area Difference(cm^2^)	2
Afán-Argüín 2023 (Afan-Arguin et al., 2023)	20females NMES(n=10) COMB(n=10)	20.5 ± 3y 22.11 ± 2.43kg/m^2^ 20.3 ± 2.32y 21.37 ± 1.13kg/m^2^	1	80%AOP+350 μs 50Hz	350 μs 50Hz	Maximal Concentric Strength (N), Maximal Eccentric Strength (N), Thigh Circumference (cm)	1
Natsume 2015 (Natsume et al., 2015)	8males MIX(n=8)	26.2 ± 2.0y 23.4 ± 3.4 kg/m^2^	20	≤50cm: 140mmHg 50-55cm: 160mmHg ≥60cm: 200mmHg+30Hz (equivalent to 5%-10% MVC)	30 Hz (equivalent to 5%-10% MVC)	Maximal Isometric Strength (N), Maximal Isokinetic Strength (N), Muscle Thickness (mm)	1
Okamura 2024 (Okamura et al., 2024)	16 males 16females NMES(n=16) COMB(n=16)	20.0 ± 1.48y 21.0 ± 2.30kg/m^2^ 21.0 ± 0.52y 21.5 ± 2.44kg/m^2^	1	70% AOP + 300 μs 80 Hz Individual Max Intensity	300 μs 80 Hz Individual Max Intensity	Abductor Hallucis Muscle Thickness(cm^2^)	1
Head 2020 (Head et al., 2020)	15males 5females MIX (n=20)	27 ± 4y 25 ± 3 kg/m^2^	4	0% 40% 60% 80% AOP + 400 μs 50 Hz Individual Max Intensity	400 μs 50 Hz Individual Max Intensity	Maximal Isometric Strength (Nm), Vastus Medialis Muscle Thickness (mm), Vastus Lateralis Muscle Thickness (mm)	1
Santiago-Pescador 2022 (Santiago-Pescador et al., 2023)	5males 15females BFR-HF(n=20) BFR-LF(n=20) NMES-HF(n=20) NMES-LF(n=20)	24.8 ± 7.0y	5	50% AOP + 350 μs 80 Hz 70% Individual Max Intensity 50% AOP + 350 μs 10 Hz 70% Individual Max Intensity	350 μs 80 Hz 70% Individual Max Intensity 350 μs 10 Hz 70% Individual Max Intensity	Maximal Isometric Strength (kg), Rectus Femoris Muscle Thickness (mm), Vastus Lateralis Muscle Thickness (mm)	2

BFR, Blood Flow Restriction; NMES, Neuromuscular Electrical Stimulation; AOP, Arterial Occlusion Pressure; Hz, Hertz; μs, Microseconds; mA, Milliampere; MVC, Maximal Voluntary Contraction; CSA, Cross-Sectional Area; RF, Rectus Femoris; VM, Vastus Medialis; VL, Vastus Lateralis; BMI, Body Mass Index; MIX, Males+ Females; COMB, BFR+NMES; HF, High Frequency; LF, Low Frequency.

Unit and Scale Notes: Muscle thickness is reported in mm unless otherwise noted; cross-sectional area (CSA) is reported in cm^2^; strength measures are reported in kg, N, or Nm as indicated; age is reported in years (y); BMI is reported in kg/m^2^; BFR pressure is reported in mmHg or as a percentage of arterial occlusion pressure (AOP); NMES parameters are reported in microseconds (μs), Hertz (Hz), or milliamperes (mA).

### 2.5 Risk of bias assessment

Two independent reviewers (SX and LHP) systematically evaluated the methodological quality of the included studies using the Cochrane Collaboration’s Risk of Bias 2.0 (ROB 2.0) tool for randomized controlled trials. This tool assesses risk of bias across five domains: (1) the randomization process; (2) deviations from intended interventions; (3) missing outcome data; (4) measurement of the outcome; and (5) selection of the reported result. Each domain was judged as “low risk of bias,” “some concerns,” or “high risk of bias” according to the ROB 2.0 handbook criteria. An overall risk of bias judgment for each study was derived by synthesizing the assessments across the five domains. Discrepancies between the two reviewers were independently reassessed by a third reviewer (ZZY) and resolved through discussion. The assessment results were used to describe study quality and potential bias risk, rather than as criteria for study inclusion or exclusion.

### 2.6 Quality assessment

The methodological quality of each included study was assessed using the modified Jadad scale. Studies with a Jadad score ≥ 4 were considered high quality, whereas those with a Jadad score < 4 were considered low quality.

### 2.7 Data analysis

Given the inherent clinical and methodological heterogeneity among the included studies, particularly regarding intervention duration and blood flow restriction (BFR) pressure prescription strategies, this study primarily employed a systematic review approach to qualitatively synthesize and descriptively analyze the extracted data.

Building upon the qualitative synthesis, an exploratory quantitative analysis was conducted to further delineate the effect trends associated with different intervention modalities. The sample size (N), mean (M), and standard deviation (SD) for both the intervention and control groups were extracted from each included study. For outcome measures with varying measurement units or assessment tools (e.g., different modalities of muscle strength testing), the standardized mean difference (SMD) was utilized as the pooled effect size. Conversely, for morphological outcomes with consistent measurement units (e.g., muscle thickness measured in millimeters), the mean difference (MD) was calculated. All quantitative results are presented with their respective 95% confidence intervals (CIs).

Statistical heterogeneity across studies was evaluated using the Chi^2^ test and the I^2^ statistic. Homogeneity was considered acceptable if I^2^ < 50% and p ≥ 0.10, in which case a fixed-effects model was used for data synthesis. Conversely, if I^2^ > 50% or p < 0.10, indicating significant inter-study heterogeneity, a random-effects model was applied. In such instances, potential sources of heterogeneity were further explored through a collinearity analysis of the intervention characteristics.

In accordance with the methodological guidelines in the Cochrane Handbook for Systematic Reviews of Interventions, formal publication bias assessments (e.g., funnel plots or Egger’s test) were not conducted, given that the number of independent data points within each sub-outcome category was fewer than ten. All data management, individual effect size calculations, and the generation of stratified exploratory forest plots were performed using R software (version 4.3.1, R Core Team). Additionally, the collinearity bubble charts illustrating multivariate intervention characteristics were custom-generated and exported using GraphPad Prism (version 10.1.2).2.8 Assessment of Evidence Quality.

The GRADE (Grading of Recommendations, Assessment, Development and Evaluation) approach was used to assess the quality of evidence for each outcome. Evidence was downgraded or upgraded based on the following five domains: risk of bias, inconsistency, indirectness, imprecision and publication bias. The quality of evidence for each outcome was rated as high, moderate, low or very low. Two reviewers (SX and LHP) independently performed the GRADE assessment, with any disagreements resolved through discussion or arbitration by a third reviewer (ZZY).

## 3 Results

### 3.1 Study selection

**Figure 1:**
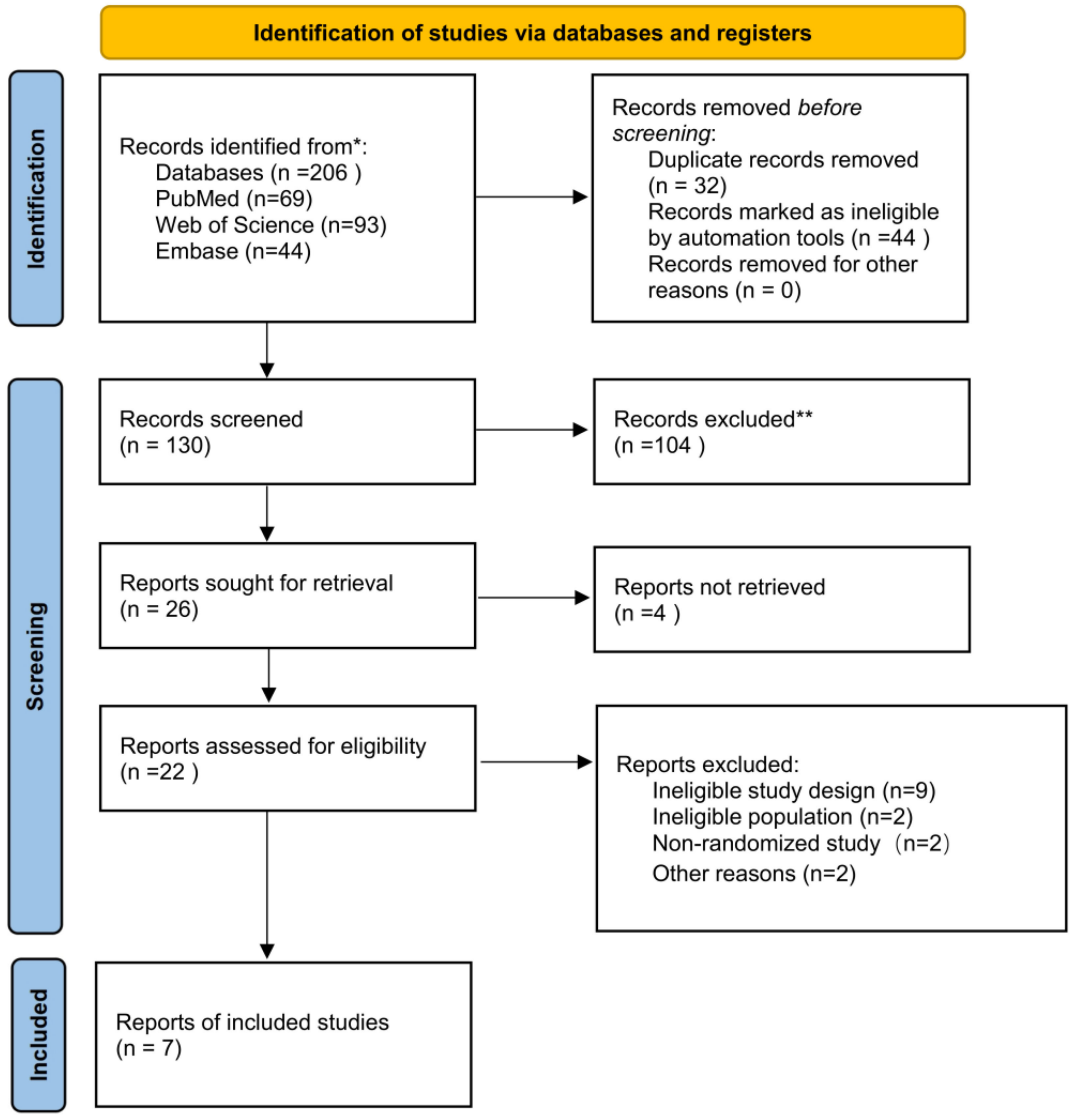
Literature inclusion screening chart. The symbol "*" indicates the databases from which the records were initially identified (PubMed, Web of Science, Embase), and "**" denotes the records excluded during the screening phase.

A total of 206 records were retrieved from the PubMed, Web of Science, and Embase databases. In accordance with Article 8 of PRISMA 2020, EndNote X9 software was used for duplicate record identification and management, with 32 duplicate records excluded; Rayyan software was adopted for preliminary screening of ineligible records, and the exclusion was only used for initial marking without subsequent manual verification. The exclusion rules of Rayyan software included two thresholds: 1) records whose titles and abstracts did not contain core keywords; 2) records that were not original research articles. A total of 44 records were excluded based on the above rules. After excluding 32 duplicate records and 44 records initially marked as ineligible by automated tools, 130 studies entered the initial screening stage. Following title and abstract screening, 104 studies that did not meet the topical relevance or abstract criteria were excluded, leaving 26 full-text articles for further assessment. Of these, four studies were excluded due to the unavailability of full texts. Ultimately, 22 studies underwent eligibility assessment, and based on the predefined inclusion and exclusion criteria, seven studies met the criteria and were included in the systematic review and quantitative synthesis (**Figure 1**).

### 3.2 Characteristics of included studies

The seven included studies comprised a total of 124 participants (74 men and 50 women), with mean ages ranging from 20.0 to 27.0 years, all of whom were healthy adults. All studies adopted randomized or crossover controlled designs, with intervention groups including BFR-NMES, NMES, and their variants with different stimulation frequencies or pressure levels. The number of intervention sessions ranged from 1 to 30. BFR pressure settings were primarily based on individual arterial occlusion pressure (AOP; 50–80%) or fixed pressures (140–350 mmHg). NMES parameters generally included pulse widths of 300–400 μs, frequencies of 30–100 Hz, and stimulation intensities reaching the maximum tolerable level for each individual. The intervention sites were mainly focused on lower-limb muscle groups (quadriceps femoris and anterior thigh muscles), with only one study targeting the abductor hallucis muscle of the lower limb. Regarding intervention modality, five studies employed static or isometric contraction protocols, while two studies used dynamic or eccentric contraction protocols. Primary outcome measures included maximal isometric or isokinetic muscle strength (N or Nm), thigh dimensions, and muscle thickness or cross-sectional area (mm, cm^2^, or g). The basic characteristics of the included studies are summarized in **Table 1**.

### 3.3 Study bias assessment results

**Figure 2:**
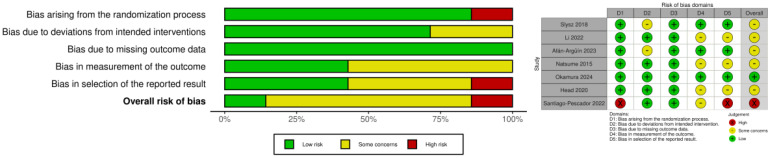
Risk of bias graph.

Seven randomized controlled trials were included in this study, and the risk of bias was assessed using the Cochrane ROB 2.0 tool, with the results presented in **Figure 2**. Overall, most studies were judged to be at low risk of bias in the domains of the randomization process, missing outcome data, and outcome measurement, with only a few studies showing some concerns regarding intervention adherence or outcome reporting. Specifically, the overall quality of the randomization process (D1) was high, as all studies except Santiago-Pescador et al. (2023) clearly reported methods of random sequence generation and allocation concealment. In the domain of deviations from intended interventions (D2), approximately one-third of the studies were rated as having “some concerns,” mainly due to the lack of complete blinding of participants or operators during intervention implementation. All studies were judged to be at low risk in the missing outcome data domain (D3), indicating good data completeness. In terms of outcome measurement (D4), some studies were rated as having “some concerns” because outcome assessors were not blinded. Selective reporting bias (D5) was judged to be at high risk in two studies, primarily due to the absence of prospective trial registration or incomplete reporting of all prespecified outcomes. The overall risk-of-bias assessment indicated that five studies were classified as having “some concerns,” one study (Okamura et al., 2024) was judged to be at low risk, and one study (Santiago-Pescador et al., 2023) was judged to be at high risk. Domain-level analysis showed that the proportion of “low risk” judgments was the highest across all bias domains (approximately 68%), followed by “some concerns” (approximately 23%), with only about 9% classified as “high risk.” In summary, while performance and detection biases were inevitable due to the nature of the intervention (lack of blinding), the risk of bias regarding randomization and data completeness was low. Therefore, the overall methodological quality was deemed acceptable for conducting the meta-analysis.

### 3.4 Quality of included studies

Assessment using the modified Jadad scale yielded scores < 4 for all included studies (**Table 1**). This low scoring was primarily driven by the inherent difficulty in double-blinding participants and therapists in electrical stimulation and tourniquet-based interventions. However, all studies employed appropriate randomization procedures3.5 Qualitative synthesis of BFR-NMES on skeletal muscle adaptations: categorized by intervention duration.

#### 3.5.1 Acute intervention effects

##### 3.5.1.1 Acute alterations in muscle strength

According to the included literature (**Table 2**, see **Supplementary Material**), the immediate effects of acute interventions on muscle strength demonstrated distinct discrepancies across different intervention modalities.

**Table 2:** Effects of acute intervention on muscle strength.

Study	Outcomes	NMES (M ± SD)			BFR-NMES (M ± SD)			Between-group
		Pre	Post	Δ (%)	Pre	Post	Δ (%)	
(Afan-Arguin et al., 2023)	MVEC	201.9 ± 63.1	220.1 ± 76.3	9.01%	212.6 ± 91.8	209.1 ± 78.5	-1.65%	-0.42
	MVCC	204.7 ± 51.1	218 ± 46.3	6.5%	215 ± 35.4	205.9 ± 41.8	-4.23%	-0.2
(Head et al., 2020)	MVIC	239.8 ± 51.3	231.5 ± 57.1	-3.46%	241.1 ± 51.1	217.8 ± 51.8	-9.66%	-0.44
(Santiago-Pescador et al., 2023)	MVIC	66.40 ± 21.31	66.15 ± 20.63	-0.38%	68.01 ± 22.19	64.33 ± 21.01	-5.41%	-0.35
	CMJ	13.36 ± 3.86	12.47 ± 3.36	-6.66%	13.73 ± 3.99	11.89 ± 3.50	-13.4%	-5.22

Immediate effects of NMES alone: The acute effects of neuromuscular electrical stimulation (NMES) alone on muscle strength were inconsistent. Afan-Arguin et al. (2023) reported that following NMES alone, maximal voluntary eccentric contraction (MVEC) and maximal voluntary concentric contraction (MVCC) increased immediately by 9.01% and 6.5%, respectively. However, two other studies reported slight immediate declines in strength performance. Head et al. (2020) observed a 3.46% reduction in maximal voluntary isometric contraction (MVIC), whereas Santiago-Pescador et al. (2023) reported a 0.38% decrease in MVIC and a 6.66% reduction in countermovement jump (CMJ) performance.

Immediate effects of BFR-NMES intervention: In contrast to the mixed findings observed with NMES alone, acute blood flow restriction combined with neuromuscular electrical stimulation (BFR-NMES) consistently resulted in immediate reductions in strength and exercise performance. Across all included reports, all performance indicators in the BFR-NMES groups declined, with reductions ranging from -1.65% (MVEC) to -13.4% (CMJ).

Between-group comparisons: For acute changes in muscle strength, all reported between-group difference values were negative (ranging from -0.20 to -5.22). This trend objectively indicates that, compared with NMES alone, acute BFR-NMES intervention induced more pronounced and consistent muscle fatigue and strength loss.

##### 3.5.1.2 Acute changes in muscle morphology

Based on the integrated findings from the included studies (**Table 3**, see **Supplementary Materials**), acute interventions resulted in immediate increases in muscle morphological indicators in both groups. However, the morphological changes induced by blood flow restriction combined with neuromuscular electrical stimulation (BFR-NMES) were more pronounced.

**Table 3:** Effects of acute intervention on muscle morphology.

Study	Outcomes	NMES (M ± SD)			BFR-NMES (M ± SD)			Between-group
		Pre	Post	Δ (%)	Pre	Post	Δ (%)	
(Afan-Arguin et al., 2023)	TC	48.50 ± 5.00	49.16 ± 5.08	1.36%	47.81 ± 3.49	48.62 ± 3.44	1.69%	0.27
(Okamura et al., 2024)	CSA(AH)	2.34 ± 0.45	2.38 ± 0.50	1.71%	1.96 ± 0.66	2.13 ± 0.68	8.67%	1.82
(Head et al., 2020)	MT(VM)	25.0 ± 2.7	25.6 ± 2.6	2.4%	24.97 ± 2.83	25.90 ± 2.87	3.72%	0.33
	MT(VL)	17.2 ± 2.8	17.9 ± 2.8	4.07%	16.83 ± 2.60	18.03 ± 3.03	7.13%	0.5
(Santiago-Pescador et al., 2023)	MT(RF)	17.31 ± 3.11	17.84 ± 3.04	3.06%	17.50 ± 3.18	19.30 ± 3.00	10.29%	0.64
	MT(VL)	22.53 ± 3.54	23.38 ± 3.64	3.77%	22.38 ± 3.72	23.81 ± 3.96	6.39%	0.58

Immediate effects of NMES alone: Neuromuscular electrical stimulation (NMES) alone was able to induce mild acute muscle morphological changes. Across the included studies, all post-intervention morphological indicators demonstrated positive increases, although the magnitude of change was relatively small. Specifically, Afan-Arguin et al. (2023) reported a 1.36% increase in thigh circumference (TC), while Okamura et al. (2024) observed a 1.71% increase in muscle cross-sectional area (CSA-AH). Regarding muscle thickness (MT), the studies by Head et al. (2020) and Santiago-Pescador et al. (2023) reported increases ranging from 2.4% to 4.07% in the vastus medialis (VM), vastus lateralis (VL), and rectus femoris (RF).

Immediate effects of BFR-NMES intervention: In comparison, acute BFR-NMES intervention induced a more substantial immediate muscle swelling effect. Except for the increase in TC reported by Afan-Arguin et al. (2023) (1.69%), which was similar to that of the NMES-alone group, all other morphological indicators demonstrated markedly greater increases than those observed with electrical stimulation alone. For example, Okamura et al. (2024) reported an 8.67% increase in CSA(AH), while Santiago-Pescador et al. (2023) observed an immediate increase of up to 10.29% in rectus femoris muscle thickness (MT-RF). Overall, the acute morphological changes in the BFR-NMES groups ranged from 1.69% to 10.29%.

Between-group comparisons: All between-group difference values related to acute muscle morphological changes were positive (ranging from 0.27 to 1.82). This objective finding clearly indicates that, compared with NMES alone, acute BFR-NMES intervention induced greater acute muscle swelling and fluid shift responses.

#### 3.5.2 Long-term intervention effects

##### 3.5.2.1 Long-term adaptations in muscle strength

In contrast to the immediate fatigue effects induced by acute interventions, long-term intervention data consistently demonstrated that blood flow restriction combined with neuromuscular electrical stimulation (BFR-NMES) provided significant advantages in promoting chronic adaptations in muscle strength (**Table 4**, see **Supplementary Materials**).

**Table 4:** Effects of long-term intervention on muscle strength.

Study	Outcomes	NMES(M ± SD)				BFR-NMES(M ± SD)				Between-group
		Pre	Post	Difference	Δ (%)	Pre	Post	Difference	Δ (%)	
(Slysz and Burr, 2018)	MVIC	141 ± 44	159 ± 43	23 ± 9	12.77%	136 ± 38	168 ± 53	32 ± 19	23.53%	0.59
(Li et al., 2023)	MVIC			20.18 ± 36.66				82.18 ± 21.64		1.97
(Natsume et al., 2015)	MVIC	287.0 ± 25.45	296.0 ± 22.62	9.0 ± 15.44	3.14%	278.0 ± 48.08	312.0 ± 73.53	34.0 ± 45.41	12.23%	0.7
	MVC90°/s	207.0 ± 33.94	204.0 ± 67.88	-3.00 ± 40.16	-1.45%	202.0 ± 45.25	212.0 ± 50.91	10.00 ± 22.20	4.95%	13
	MVC180°/s	159.0 ± 36.77	159.0 ± 25.45	0.00 ± 17.76	0%	158.0 ± 42.42	171.0 ± 42.42	13.00 ± 18.97	8.23%	13

Long-term effects of NMES alone: Long-term NMES intervention alone produced relatively limited improvements in muscle strength, and the effects varied across different measurement indicators. For maximal voluntary isometric contraction (MVIC), Slysz and Burr (2018) and Natsume et al. (2015) reported increases of 12.77% and 3.14%, respectively. Li et al. (2023) also observed positive changes in MVIC in the NMES-alone group (pre-post difference: 20.18 ± 36.66). However, regarding isokinetic strength outcomes, Natsume et al. (2015) found a slight 1.45% decrease in MVC at 90°/s (MVC90°/s) in the NMES-alone group, whereas MVC at 180°/s (MVC180°/s) showed no obvious change (0%).

Long-term effects of BFR-NMES intervention: In contrast, following long-term BFR-NMES intervention, all muscle strength outcomes consistently demonstrated positive improvements, with substantially greater increases than those observed in the NMES-alone groups. In the study by Slysz and Burr (2018), the MVIC of the BFR-NMES group increased by as much as 23.53%. Natsume et al. (2015) also reported comprehensive improvements in several indicators in the BFR-NMES group, including MVIC (12.23%), MVC90°/s (4.95%), and MVC180°/s (8.23%). Furthermore, in the study by Li et al. (2023), the pre-post MVIC difference in the BFR-NMES group reached 82.18 ± 21.64, which was substantially greater than that observed in the NMES-alone group.

Between-group comparisons: All reported between-group difference values in the included studies were positive (ranging from 0.59 to 13). This objective data trend confirms that long-term BFR-NMES intervention was significantly more effective than NMES alone in improving muscle strength.

##### 3.5.2.2 Long-term adaptations in muscle morphology

Consistent with the trends observed in long-term muscle strength adaptations, long-term combined intervention strategies also demonstrated superior effects in promoting chronic muscle morphological adaptations, such as muscle hypertrophy (**Table 5**, see **Supplementary Materials**).

**Table 5:** Effects of long-term intervention on muscle morphology.

Study	Outcomes	NMES(M ± SD)				BFR-NMES(M ± SD)				Between-group
		Pre	Post	Difference	Δ (%)	Pre	Post	Difference	Δ (%)	
(Slysz and Burr, 2018)	MM	9311 ± 2551	9391 ± 2427	23 ± 9	0.86%	9139 ± 2284	9234 ± 2256	96 ± 225	1.04%	0.05
(Li et al., 2023)	CSA(RF)			0.11 ± 0.19				1.13 ± 0.17		5.42
(Natsume et al., 2015)	MT(Quadriceps)	47.8 ± 9.05	47.7 ± 9.90	-0.10 ± 3.11	-0.21%	47.6 ± 9.05	47.8 ± 9.62	0.20 ± 3.01	0.42%	0.09

Long-term effects of NMES alone: Long-term neuromuscular electrical stimulation (NMES) training alone showed relatively limited effects on promoting muscle hypertrophy and may even result in stagnation. Slysz and Burr (2018) observed only a slight 0.86% increase in muscle mass (MM) following intervention. Li et al. (2023) similarly reported only a minimal positive change in rectus femoris cross-sectional area (CSA-RF) (pre-post difference: 0.11 ± 0.19). Furthermore, Natsume et al. (2015) found that following long-term NMES intervention alone, quadriceps muscle thickness (MT-Quadriceps) not only failed to increase but instead demonstrated a slight decrease (-0.21%).

Long-term effects of BFR-NMES intervention: In comparison, long-term blood flow restriction combined with electrical stimulation (BFR-NMES) demonstrated more consistent positive effects on muscle morphological adaptations. During the same intervention period, Slysz and Burr (2018) reported a 1.04% increase in muscle mass in the BFR-NMES group. Li et al. (2023) found that the absolute increase in rectus femoris cross-sectional area in the BFR-NMES group (1.13 ± 0.17) was substantially greater than that observed in the NMES-alone group. In addition, unlike the slight decline observed in the electrical stimulation-alone group, Natsume et al. (2015) reported a 0.42% increase in quadriceps muscle thickness in the BFR-NMES group.

Between-group comparisons: All between-group difference values related to long-term muscle morphological adaptations in the included studies were positive (ranging from 0.05 to 5.42). This objective finding clearly confirms that, from a long-term intervention perspective, the combined application of blood flow restriction and electrical stimulation (BFR-NMES) was more effective than electrical stimulation alone in inducing muscle hypertrophy and promoting positive morphological adaptations.

### 3.6 Exploratory analysis

**Figure 3:**
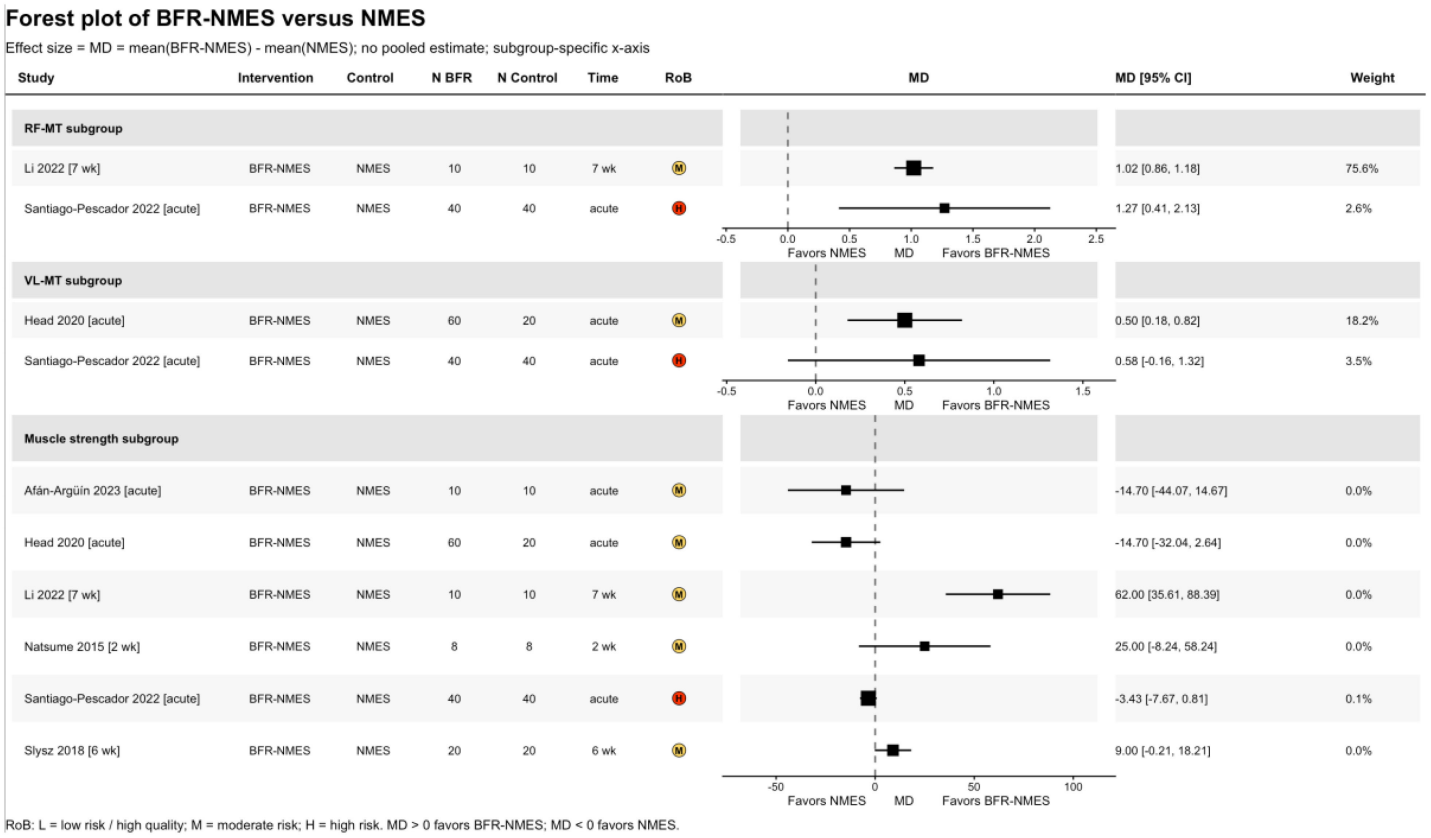
Exploratory forest plot mapping raw effect trends across sub-outcomes without a pooled estimate.

To further visually illustrate the effect trends between BFR-NMES and NMES alone, an exploratory data visualization analysis was conducted on studies with available quantitative data. However, considering the inherent clinical and methodological heterogeneity among the included studies in terms of intervention duration, pressure strategies, and outcome measurements, a full quantitative statistical pooling was not performed in this section. Instead, the present study first conducted a collinearity assessment of study characteristics and subsequently integrated the effect sizes, intervention duration (Time), and risk of bias (RoB) from each independent study into an exploratory integrated forest plot (**Figure 3**). This approach aimed to objectively reveal the underlying distribution patterns of data points and potential sources of heterogeneity across different intervention characteristics through visual stratification.

#### 3.6.1 Collinearity analysis of study characteristics

**Figure 4:**
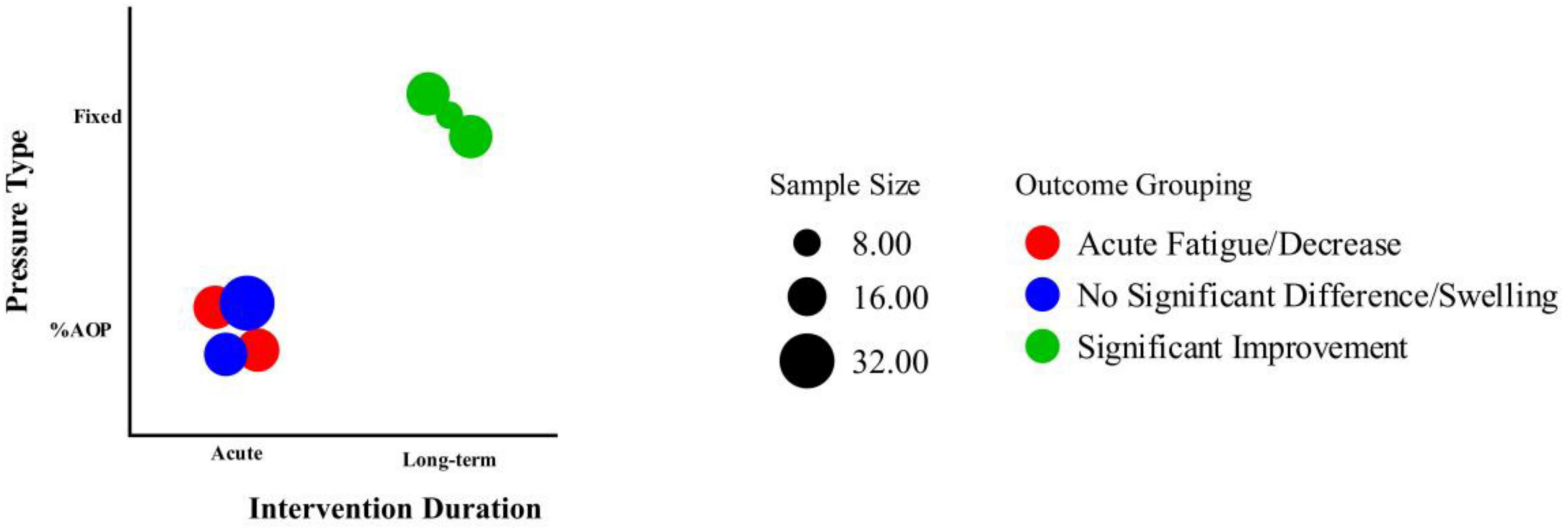
Bubble chart illustrating the methodological collinearity between intervention duration and pressure type across included trials.

Through bubble plot visualization analysis of intervention duration (acute vs. long-term), blood flow restriction pressure-setting strategy (%AOP vs. fixed pressure), and final outcome categories (**Figure 4**), the present study identified substantial collinearity among variables. The analysis demonstrated that most acute intervention studies adopted percentage arterial occlusion pressure (%AOP)-based pressure settings, and their corresponding outcomes were primarily clustered around “acute fatigue/performance decline” (red cluster) or “no significant difference/acute swelling” (blue cluster). In contrast, long-term intervention studies were highly concentrated in the use of “fixed pressure” strategies and consistently resulted in “significant improvement” outcomes (green cluster). This strong coupling between intervention duration and pressure-setting strategy suggests that pooling all studies into a single quantitative effect size synthesis would introduce substantial heterogeneity. Therefore, subsequent quantitative findings should be interpreted with caution.

#### 3.6.2 Results of the exploratory meta-analysis

To more intuitively present the raw data trends and potential sources of heterogeneity across different outcome measures in the included studies, an exploratory integrated forest plot was generated (**Figure 3**). Given the methodological differences identified in the previous collinearity analysis, no overall pooled effect size was calculated in this section. Instead, subgroup visualizations were presented according to outcome categories. Intervention duration (acute vs. long-term) and risk of bias (RoB) were annotated in the figure to facilitate interpretation of differences in effect direction across studies. These quantitative findings should be regarded only as preliminary hypothesis-generating evidence intended to support the qualitative conclusions presented above.

##### 3.6.2.1 Muscle strength

In the muscle strength subgroup (**Figure 3**, Muscle strength subgroup), the forest plot clearly and visually demonstrated the decisive influence of intervention duration on effect direction. The mean differences (MDs) reported in the three acute intervention studies (Afan-Arguin et al., 2023; Head et al., 2020; Santiago-Pescador et al., 2023) all favored the NMES-alone group (MD < 0), objectively reflecting the immediate strength reduction and pronounced fatigue induced by acute BFR-NMES intervention. In contrast, the data points from the three long-term intervention studies (Li et al., 2023, Natsume et al., 2015, and Slysz and Burr, 2018) consistently favored the BFR-NMES group (MD > 0), among which Li et al. (2023) reported an especially pronounced positive improvement in strength. This distinctly opposing visual distribution further supports the conclusions of the qualitative analysis, indicating that intervention duration is a critical confounding factor regulating the direction of muscle strength adaptations.

##### 3.6.2.2 VL thickness

Regarding acute changes in vastus lateralis thickness (**Figure 3**, VL-MT subgroup), the two included acute intervention studies, Head et al. (2020) and Santiago-Pescador et al. (2023), demonstrated highly consistent trends. The data points and 95% confidence intervals from both studies were located on the right side of the line of no effect, favoring the BFR-NMES group. At the quantitative level, these findings visually support the notion that BFR-NMES possesses a more consistent advantage than NMES alone in inducing acute muscle swelling and fluid shifts.

##### 3.6.2.3 RF morphology

In the rectus femoris morphology subgroup (**Figure 3**, RF-MT subgroup), both the long-term intervention study by Li et al. (2023) (7 weeks) and the acute intervention study by Santiago-Pescador et al., (2023) demonstrated superior outcomes for BFR-NMES compared with NMES alone (MD > 0). However, as indicated by both the forest plot and the risk of bias (RoB) assessment, the two studies differed fundamentally in intervention duration and study design, and Santiago-Pescador et al., (2023) was associated with a higher risk of bias. Although the effect directions appeared superficially consistent, direct statistical pooling was not performed due to the fundamentally different underlying physiological mechanisms.

### 3.7 GRADE assessment of evidence quality

The GRADE approach was used to assess the quality of evidence for the main outcomes, with results summarized in **Table 6**. For muscle strength (overall), the quality of evidence was rated as very low, primarily due to serious concerns regarding risk of bias and inconsistency, as well as imprecision related to the relatively small sample size. For the acute intervention subgroup, the quality of evidence was moderate; despite risk of bias concerns, consistency across studies was high and effect estimates were precise. For the long-term intervention subgroup, the quality of evidence was low, downgraded due to imprecision from the small sample size and moderate heterogeneity. For vastus lateralis thickness, the quality of evidence was moderate, with high consistency between studies but imprecision due to the limited number of studies and small sample size.

**Table 6:** GRADE assessment of evidence quality.

BFR-NMES versus NMES alone for skeletal muscle hypertrophy and strength in healthy adults										
Patient or population: Healthy adults (targeting skeletal muscle hypertrophy and strength improvement) Settings: Clinical or research setting for healthy adult exercise interventions Intervention: Blood Flow Restriction Neuromuscular Electrical Stimulation (BFR-NMES)										
Outcomes	Study design	Risk of bias	Inconsistency	Indirectness	Imprecision	Publication bias	No of Participants (studies)	Effect estimate		Quality of the evidence (GRADE)
Muscle strength	RCT	Serious	Serious	Not serious	Serious	Undetected	256 (6 studies)		SMD = 0.24 95% CI [-0.37,0.85]; I^2^ = 79%	⊕⊝⊝⊝ **very low**
Muscle strength (Acute intervention)	RCT	Serious	Not serious	Not serious	Not serious	Undetected	180 (3 studies)		SMD = -0.39 95% CI [-0.70, -0.08]; I^2^ = 0%	⊕⊕⊕⊝ **moderate**
Muscle strength (Long-term intervention)	RCT	Serious	Not serious	Not serious	Serious	Undetected	76 (3 studies)		SMD = 1.00 95% CI [0.21, 1.80]; I^2^ = 57%	⊕⊕⊝⊝ **low**
Vastus lateralis thickness	RCT	Not serious	Not serious	Not serious	Serious	Undetected	160 (2 studies)		MD = 0.51mm 95% CI [0.22, 0.81]; I^2^ = 0%	⊕⊕⊕⊝ **moderate**

GRADE Working Group grades of evidence.

High quality: Further research is very unlikely to change our confidence in the estimate of effect.

Moderate quality: Further research is likely to have an important impact on our confidence in the estimate of effect and may change the estimate.

Low quality: Further research is very likely to have an important impact on our confidence in the estimate of effect and is likely to change the estimate.

Very low quality: We are very uncertain about the estimate.

## 4 Discussion

This systematic review, combined with an exploratory quantitative analysis, investigated the acute responses and long-term adaptations of blood flow restriction combined with neuromuscular electrical stimulation (BFR-NMES) compared with NMES alone on skeletal muscle morphology and strength in healthy adults. Preliminary evidence suggests that BFR-NMES may demonstrate greater potential than NMES alone in inducing acute muscle fluid shifts (e.g., immediate increases in vastus lateralis thickness) and promoting positive long-term muscle morphological adaptations.

However, when all studies were quantitatively pooled together, no statistically significant differences were observed in muscle strength or rectus femoris thickness. Importantly, the extremely high heterogeneity revealed the critical moderating role of intervention duration. Long-term interventions tended to promote chronic adaptations in muscle strength, whereas acute interventions consistently resulted in greater immediate strength reductions, reflecting neuromuscular fatigue.

From a mechanistic perspective, BFR-NMES is believed to exert synergistic effects through multiple physiological pathways. First, NMES possesses a unique motor unit recruitment pattern. Unlike voluntary contractions, which follow the Henneman size principle, NMES recruits motor units in a nonselective and synchronous manner, preferentially activating high-threshold fast-twitch muscle fibers (type II fibers) while bypassing the gradual recruitment sequence (Rice et al., 2023; Chen et al., 2025). Second, BFR partially restricts venous return, thereby creating a localized hypoxic environment within the muscle and promoting the accumulation of metabolic by-products such as lactate and inorganic phosphate (Günaydın et al., 2026). This hypoxic condition facilitates earlier recruitment of type II fibers, thereby synergizing with the preferential type II fiber activation induced by NMES (Liu et al., 2024). The synergistic interaction between the two techniques primarily occurs at the level of metabolic stress. BFR prolongs the retention time of metabolic by-products within the muscle through blood flow restriction, whereas the synchronous contractions induced by NMES generate substantial amounts of metabolic metabolites. Their combination may therefore create a metabolic stress environment far greater than that produced by either intervention alone. Although the mechanical tension generated by BFR-NMES is relatively limited, this pronounced metabolic stress has been shown to be sufficient to independently stimulate protein synthesis and promote positive muscle morphological adaptations through activation of signaling pathways such as mTOR and MAPK (Natsume et al., 2019).

One particularly important methodological finding emerging from the current evidence is the complete collinearity (confounding) between intervention duration and BFR pressure prescription strategy. All included long-term studies adopted fixed-pressure protocols, whereas all acute studies used individualized pressure based on arterial occlusion pressure (%AOP). Therefore, based on the currently limited literature, the present study was unable to disentangle and confirm whether the observed improvements in muscle strength and morphology were primarily driven by intervention duration (training period) or by the specific pressure strategy employed. This substantially limits our deeper understanding of the underlying mechanisms. Future studies should attempt to manipulate these parameters independently. For example, individualized %AOP pressure strategies could be applied in long-term interventions, or fixed-pressure protocols could be tested in acute designs, in order to clarify their respective contributions and optimize the clinical prescription of BFR-NMES.

### 4.1 Limitation

While this meta-analysis provides novel insights into the efficacy of BFR-NMES, several limitations must be acknowledged when interpreting the results.

First, and most importantly, there was a complete confounding between intervention duration and BFR pressure prescription, which prevented independent evaluation of these two key variables.

Second, sensitivity analyses based on overall ROB 2.0 rating and the exclusion of Okamura et al., 2024 were conducted; these analyses confirmed the robustness of the primary findings. However, given the limited number of studies, the power of these sensitivity analyses remains constrained, and the results should be interpreted with caution.

Third, the included studies exhibited considerable heterogeneity in NMES parameters (frequency range: 30–100 Hz, pulse width range: 300–400 μs, stimulation intensity ranging from fixed mA to individual maximum tolerance), parameters that significantly influence motor unit recruitment depth and metabolic demand. The sample size in this study was insufficient to conduct dose-response subgroup analyses or meta-regression. Future studies should consider standardizing or systematically manipulating these key parameters in their experimental designs to assess their independent contributions to the effects of BFR-NMES.

Fourth, the limited number of included studies and the small total sample size constrained statistical power. With only seven studies and 124 participants, meta-regression analyses to explore key mechanistic moderators (e.g., stimulation frequency, intensity, sex differences) were not feasible. Furthermore, the heterogeneity in participant sex composition across studies further complicated the interpretation of pooled estimates, as sex-specific responses could not be examined.

Finally, the methodological quality of the included studies was moderate, as reflected by low modified Jadad scores. This limitation primarily stems from the inherent difficulty of blinding participants and therapists in BFR and NMES interventions. While randomization and outcome completeness were generally adequate, performance and detection bias cannot be fully excluded, particularly for strength outcomes influenced by discomfort and motivation.

## Conclusion

1. Regarding Muscle Hypertrophy: The available evidence, although derived from a limited number of studies, consistently suggests that BFR-NMES may offer additional benefits for increasing vastus lateralis muscle thickness. However, the findings for rectus femoris hypertrophy should be interpreted with caution due to substantial heterogeneity. These preliminary results provide a rationale for conducting larger, confirmatory trials to explore muscle-specific hypertrophic responses.
2. Regarding Muscle Strength: Although no significant overall advantage was observed for maximal isometric strength, subgroup analyses indicated that BFR-NMES was more favorable for strength adaptation during long-term interventions, whereas NMES alone showed an advantage in acute interventions. This advantage in acute settings likely reflects lower immediate fatigue induced by NMES alone and should not be interpreted as direct evidence of its superior long-term adaptation compared to BFR-NMES.

In summary, the current evidence base, while promising and internally consistent for certain outcomes, is derived from a small pool of studies with limited sample sizes. Therefore, these findings should be regarded as preliminary and hypothesis-generating. They are intended to inform the design of future high-quality randomized controlled trials rather than to serve as definitive recommendations for practice.

## Data Availability

The original contributions presented in the study are included in the article/**Supplementary Material**. Further inquiries can be directed to the corresponding authors.

## Glossary

1RM: One-Repetition Maximum
AOP: Arterial Occlusion Pressure
BFR: Blood Flow Restriction
BFR-NMES: Blood Flow Restriction combined with Neuromuscular Electrical Stimulation
BMI: Body Mass Index
CI: Confidence Interval
COMB: Combined Group (BFR + NMES)
CSA: Cross-Sectional Area
EMS: Electrical Muscle Stimulation
FES: Functional Electrical Stimulation
HF: High Frequency
Hz: Hertz
LF: Low Frequency
mA: Milliampere
MAPK: Mitogen-Activated Protein Kinase
MD: Mean Difference
MIX: Males + Females
mTOR: Mammalian Target of Rapamycin
MVC: Maximal Voluntary Contraction
N: Newton (unit of force)
Nm: Newton-meter (unit of torque)
NMES: Neuromuscular Electrical Stimulation
PICOS: Population, Intervention, Comparison, Outcome, Study design
PRISMA: Preferred Reporting Items for Systematic Reviews and Meta-Analyses
RF: Rectus Femoris
ROB 2.0: Risk of Bias 2.0
SMD: Standardized Mean Difference
VL: Vastus Lateralis
VM: Vastus Medialis
μs: Microseconds.
