# Supplementary material for: Effects of combined blood flow restriction and neuromuscular electrical stimulation versus neuromuscular electrical stimulation alone on skeletal muscle hypertrophy and strength in adults: a systematic review
Source: Front Physiol. 2026 Jun 10;17:1797485. doi: 10.3389/fphys.2026.1797485 (PMC13290688; doi:10.3389/fphys.2026.1797485)
Supplement: Supplementary file 1 [file Table1.docx]

Table2. Effects of Acute Intervention on Muscle Strength

| Study | Outcomes | NMES(M±SD) | | | BFR-NMES(M±SD) | | | Between-group |
| --- | --- | --- | --- | --- | --- | --- | --- | --- |
|  |  | Pre | Post | Δ (%) | Pre | Post | Δ (%) |  |
| Afán-Argüín 2023 | MVEC | 201.9±63.1 | 220.1±76.3 | 9.01% | 212.6±91.8 | 209.1±78.5 | -1.65% | -0.42 |
|  | MVCC | 204.7±51.1 | 218±46.3 | 6.5% | 215±35.4 | 205.9±41.8 | -4.23% | -0.2 |
| Head 2020 | MVIC | 239.8±51.3 | 231.5±57.1 | -3.46% | 241.1±51.1 | 217.8±51.8 | -9.66% | -0.44 |
| Santiago-Pescador 2022 | MVIC | 66.40±21.31 | 66.15±20.63 | -0.38% | 68.01±22.19 | 64.33±21.01 | -5.41% | -0.35 |
|  | CMJ | 13.36±3.86 | 12.47±3.36 | -6.66% | 13.73±3.99 | 11.89±3.50 | -13.4% | -5.22 |

Table3. Effects of Long-Term Intervention on Muscle Strength

| Study | Outcomes | NMES(M±SD) | | | | BFR-NMES(M±SD) | | | | Between-group |
| --- | --- | --- | --- | --- | --- | --- | --- | --- | --- | --- |
|  |  | Pre | Post | Difference | Δ (%) | Pre | Post | Difference | Δ (%) |  |
| Slysz 2018 | MVIC | 141±44 | 159±43 | 23±9 | 12.77% | 136±38 | 168±53 | 32±19 | 23.53% | 0.59 |
| Li 2022 | MVIC |  |  | 20.18±36.66 |  |  |  | 82.18±21.64 |  | 1.97 |
| Natsume 2015 | MVIC | 287.0±25.45 | 296.0±22.62 | 9.0±15.44 | 3.14% | 278.0 ± 48.08 | 312.0±73.53 | 34.0±45.41 | 12.23% | 0.7 |
|  | MVC90°/s | 207.0±33.94 | 204.0±67.88 | -3.00±40.16 | -1.45% | 202.0 ± 45.25 | 212.0±50.91 | 10.00±22.20 | 4.95% | 13 |
|  | MVC180°/s | 159.0±36.77 | 159.0±25.45 | 0.00±17.76 | 0% | 158.0 ± 42.42 | 171.0±42.42 | 13.00±18.97 | 8.23% | 13 |

Table4. Effects of Acute Intervention on Muscle Morphology

| Study | Outcomes | NMES(M±SD) | | | BFR-NMES(M±SD) | | | Between-group |
| --- | --- | --- | --- | --- | --- | --- | --- | --- |
|  |  | Pre | Post | Δ (%) | Pre | Post | Δ (%) |  |
| Afán-Argüín 2023 | TC | 48.50±5.00 | 49.16±5.08 | 1.36% | 47.81±3.49 | 48.62±3.44 | 1.69% | 0.27 |
| Okamura 2024 | CSA(AH) | 2.34±0.45 | 2.38±0.50 | 1.71% | 1.96±0.66 | 2.13±0.68 | 8.67% | 1.82 |
| Head 2020 | MT(VM) | 25.0±2.7 | 25.6±2.6 | 2.4% | 24.97±2.83 | 25.90±2.87 | 3.72% | 0.33 |
|  | MT(VL) | 17.2±2.8 | 17.9±2.8 | 4.07% | 16.83±2.60 | 18.03±3.03 | 7.13% | 0.5 |
| Santiago-Pescador 2022 | MT(RF) | 17.31±3.11 | 17.84±3.04 | 3.06% | 17.50±3.18 | 19.30±3.00 | 10.29% | 0.64 |
|  | MT(VL) | 22.53±3.54 | 23.38±3.64 | 3.77% | 22.38±3.72 | 23.81±3.96 | 6.39% | 0.58 |

Table5. Effects of Long-Term Intervention on Muscle Morphology

| Study | Outcomes | NMES(M±SD) | | | | BFR-NMES(M±SD) | | | | Between-group |
| --- | --- | --- | --- | --- | --- | --- | --- | --- | --- | --- |
|  |  | Pre | Post | Difference | Δ (%) | Pre | Post | Difference | Δ (%) |  |
| Slysz 2018 | MM | 9311±2551 | 9391±2427 | 23±9 | 0.86% | 9139±2284 | 9234±2256 | 96±225 | 1.04% | 0.05 |
| Li 2022 | CSA(RF) |  |  | 0.11±0.19 |  |  |  | 1.13±0.17 |  | 5.42 |
| Natsume 2015 | MT(Quadriceps) | 47.8±9.05 | 47.7±9.90 | -0.10±3.11 | -0.21% | 47.6±9.05 | 47.8±9.62 | 0.20±3.01 | 0.42% | 0.09 |
